# Update on Paraneoplastic Cerebellar Degeneration

**DOI:** 10.3390/brainsci11111414

**Published:** 2021-10-26

**Authors:** Philipp Alexander Loehrer, Lara Zieger, Ole J. Simon

**Affiliations:** 1 Department of Neurology, Philipps-University Marburg, 35043 Marburg, Germany; zieger@med.uni-marburg.de (L.Z.); ole.simon@uni-marburg.de (O.J.S.); 2 Department of Neurology, University Hospital Gießen and Marburg, 35043 Marburg, Germany

**Keywords:** paraneoplastic cerebellar degeneration, immune-mediated cerebellar ataxias, paraneoplastic syndromes, onconeuronal antibodies, Yo-antibody, Tr/DNER-antibody, mGluR1-antibody

## Abstract

**Purpose of review:** To provide an update on paraneoplastic cerebellar degeneration (PCD), the involved antibodies and tumors, as well as management strategies. **Recent findings:** PCD represents the second most common presentation of the recently established class of immune mediated cerebellar ataxias (IMCAs). Although rare in general, PCD is one of the most frequent paraneoplastic presentations and characterized clinically by a rapidly progressive cerebellar syndrome. In recent years, several antibodies have been described in association with the clinical syndrome related to PCD; their clinical significance, however, has yet to be determined. The 2021 updated diagnostic criteria for paraneoplastic neurologic symptoms help to establish the diagnosis of PCD, direct cancer screening, and to evaluate the presence of these newly identified antibodies. Recognition of the clinical syndrome and prompt identification of a specific antibody are essential for early detection of an underlying malignancy and initiation of an appropriate treatment, which represents the best opportunity to modulate the course of the disease. As clinical symptoms can precede tumor diagnosis by years, co-occurrence of specific symptoms and antibodies should prompt continuous surveillance of the patient. **Summary:** We provide an in-depth overview on PCD, summarize recent findings related to PCD, and highlight the transformed diagnostic approach.

## 1. Introduction

Paraneoplastic cerebellar degeneration (PCD) is a rare disorder but represents the second most frequent paraneoplastic presentation, as well as the second most common immune-mediated cerebellar ataxia (IMCA) [1,2,3]. The first documentation of a patient with PCD originates from Brouwer, who described the association of cerebellar ataxia with ovarian cancer in 1919 [4]. Since this first description, several important discoveries have been made. In 1983, Greenlee and Brashear were the first to describe the association between PCD and antibodies directed against cerebellar Purkinje cells, also known as Yo-antibodies, in a patient with ovarian carcinoma [5]. This landmark study demonstrated the autoimmune nature of PCD and paved the way for the discovery of further antibodies in patients with PCD such as Hu-, Ri-, and Ma2-antibodies and their association with specific malignancies [1]. Based on these findings, PCD was defined as a remote effect of cancer with an autoimmune pathogenesis [6]. The autoimmune response is thought to be elicited when proteins restricted to immune privileged neurons are presented by the underlying malignancy [7,8]. In general, two different mechanisms seem to be important for the development of autoimmunity in PCD. In the majority of patients with PCD, cytotoxic T-cell responses seem to play a crucial role [8,9,10]. Associated antibodies are directed against intracellular antigens and may not be directly pathogenic but rather biomarkers for the condition [6]. In some patients with PCD, however, antibodies against neural cell surface or synaptic proteins, e.g., P/Q-type voltage-gated calcium channels-(VGCC) and metabotropic glutamate receptor 1-(mGluR1) antibodies, can be detected [11,12,13]. These antibodies are thought to be directly pathogenic, as they cause receptor blockage and internalization which results in symptoms such as cerebellar ataxia [14]. Patients typically present with a rapidly progressive cerebellar syndrome, which is defined by progression to pancerebellar dysfunction within three months [8]. Clinical presentation and antibody detection can precede the diagnosis of an associated cancer in 60 to 70% [15,16,17]. Furthermore, a strong association between specific antibodies, neurologic syndrome, and cancer type exists [8]. Therefore, the combination of clinical symptoms and the presence of a specific antibody should direct the cancer search. Malignancies typically associated with PCD are small cell lung cancer (SCLC), gynecologic and breast cancer, as well as Hodgkin lymphoma [18]. By definition, diagnosis of PCD requires the exclusion of a direct (e.g., metastasis) or indirect (e.g., coagulopathy) cancer involvement as well as the exclusion of other metabolic, iatrogenic, or infectious causes [3,6]. Diagnostic criteria and screening recommendations have been published, which should guide clinical decision making when a patient presents with a rapidly progressive cerebellar syndrome [6]. When PCD is suspected, treatment should be initiated as soon as possible and includes acute immunotherapy, oncologic treatment, and maintenance immunotherapy. Outcome is typically poor, but differences exist, as patients presenting with antibodies against cell surface/synaptic proteins respond well to acute immunotherapy [19]. 

Over the past years, several new antibodies that associate with a rapidly progressive cerebellar syndrome have been identified [6]. Furthermore, new diagnostic criteria to diagnose paraneoplastic syndromes including PCD, as well as a new classification on IMCAs, have been published [6,20]. This review gives an in-depth overview on PCD, summarizes recent findings related to PCD, and highlights the transformed diagnostic approach. 

## 2. Principles of Autoimmunity

The cerebellum is a frequent target of autoimmune reactions and paraneoplastic autoimmunity in particular. The pathogenesis is attributed to an autoimmune response, which is elicited when proteins restricted to immune privileged neurons are presented by the underlying malignancy [7,8]. In about 80% of the patients presenting with PCD, neuronal antibodies can be detected [21]. In principle, these antibodies are classified according to the cellular localization of their target antigens; namely, intracellular, cell-surface, or synaptic [22]. Antibodies targeting intracellular structures are usually not considered directly pathogenic. Rather, they are regarded as biomarkers of a predominantly cell-mediated cytotoxic process, although the pathogenic role of some antibodies targeting intracellular structures is still a matter of debate [1,6,22]. In this regard, Yo- and Hu-antibodies have been studied extensively. Both antigens are expressed within the tumor, as well as neuronal cells, and the respective antibodies are thus termed onconeural antibodies [1]. Transferring these antibodies to rodents did not induce ataxic symptoms in several studies and the authors concluded that the antibodies do not have a direct pathogenic effect [23,24]. Furthermore, Carpentier et al. showed that Hu-antibodies developed when mice were immunized with Hu, but no neurological symptoms evolved [25]. This is in line with the notion that Hu-antibodies can be detected in 20% of patients with SCLC, but less than 0.01% of SCLC patients develop a paraneoplastic neurologic syndrome (PNS) [26]. On the other hand, Yo-antibodies are internalized by Purkinje-cells and impede interaction between cerebellar degeneration related protein 2 (cdr2) with the transcription factor c-Myc, which possibly results in disruption of cell cycle signaling [27]. Similarly, disruption of cdr2-interaction with mortality factor-like proteins might induce apoptosis of Purkinje-cells [28]. Therefore, a possible pathogenic effect of Yo-antibodies is discussed. This assumption is opposed by the more frequently invoked hypothesis that PCD is caused by cell-mediated cytotoxic processes [20]. In both, Yo- and Hu-antibody positive patients, high levels of cdr2- or Hu-specific T-cells are present in the blood and cerebrospinal fluid (CSF) [29,30]. Furthermore, the cerebellum is mainly infiltrated by CD3+ and CD8+ T-cells, which seem to be antigen-driven and not attracted non-specifically by a proinflammatory environment [31,32,33]. It has therefore been suggested that PCD is primarily mediated via a CD8+ T-cell immune response toward an autoantigen which is also recognized by onconeural antibodies [1]. 

Antibodies targeting cell-surface proteins, on the other hand, are thought to have a direct pathogenic effect. In the context of PCD, VGCC- and mGluR1-antibodies are relevant autoantibodies targeting cell-surface proteins and synaptic proteins, respectively [1,22]. Calcium channels targeted by VGCC-antibodies rest on the membrane of Purkinje-cells and are responsible for calcium homeostasis [14]. They therefore play a relevant role in cell function and survival [14]. Binding of VGCC-antibodies to its target leads to channel dysfunction and internalization, whereby passive transfer of VGCC-antibodies to mice caused cerebellar ataxia [14,26]. Autopsy studies demonstrated that patients with PCD and VGCC-antibodies have a diffuse loss of Purkinje cells, depletion of VGCC, and binding of VGCC-antibodies to the remaining channels [26,34]. Therefore, VGCC-antibodies are thought to have a direct pathogenic effect.

Similarly, antibodies targeting mGluR1 are thought to be responsible for the ataxic syndrome seen in mGluR1-anitbody positive PCD. mGluR1 is important for rapid signal transmission in the cerebellum as it regulates calcium signaling in Purkinje cell dendritic spines [35]. Injection of mGluR1-antibodies into the cerebellum of mice caused reduced compensatory eye movements and blocked induction of long-term depression, which is important for motor learning [13]. The authors concluded that mGluR1-antibodies cause cerebellar ataxia via a combination of rapid effects on acute and plastic Purkinje cell responses and degenerative effects [13]. The exact mechanism resulting in degeneration of Purkinje cells, however, remains a matter of debate [13]. 

## 3. Epidemiology

Paraneoplastic cerebellar degeneration is rare in general but constitutes the second most frequent paraneoplastic presentation as well as the second most common immune-mediated cerebellar ataxia [1,2,3]. Due to the rarity of the disease, extensive epidemiological studies are lacking but evidence from single-centers and population-based studies on PNS and progressive ataxias exist [2,3,36,37].

A recent population-based study in northern Italy reported an incidence of paraneoplastic syndromes of 0.89/100,000 person-years [3]. Among 89 patients with definite PNS, identified over a study period of nine years (2009–2017), 28 (31.5%) had limbic encephalitis, 25 (28.1%) had PCD, and 18 (20.2%) had encephalomyelitis. The median age of patients was 68 (range 26–90) and 52% of identified patients were female [3]. Hébert et al. reported a lower incidence rate for PNS of 0.41/100,000 person-years in a French population-based study, which was conducted between 2016 and 2018 [36]. These differences in crude incidence rates were attributed to the increased challenge of achieving exhaustiveness in a larger population [36]. However, both studies reported a year-to-year increase in incidence of PNS which might reflect the increased recognition of these disorders [3,36]. 

Pertaining to the prevalence of cerebellar ataxias and PCD in particular, there have been few large-scale studies only. Hadjivassiliou et al. reported prevalence-levels of different etiologies of progressive ataxias within a large single center cohort in the United Kingdom [2]. Within 1500 patients assessed for ataxia, 1205 cases (80%) had no family history of ataxia and were classified as sporadic. Among these sporadic cases, 32% were classified as IMCA. Gluten ataxia was the commonest IMCA accounting for 25% of sporadic ataxias, followed by PCD (3%), anti-GAD-associated ataxia (2%), postinfectious cerebellitis (PIC, 1%), and opsoclonus myoclonus ataxia (<1%) [2]. Describing the different etiologies of sporadic late-onset cerebellar ataxias within a prospective observational study in 80 patients presenting to a tertiary-care center in France, Gebus et al. reported comparable prevalence rates, including two cases of PCD (3%) and one case of PIC (1%) [38]. In a Korean population, prevalence rates for IMCAs and PCD in particular differed [37]. Within a single-center cohort of 820 patients presenting with cerebellar ataxia, 684 (83%) had no family history of ataxia. Among these non-familial ataxias, 3% could be classified as IMCA, with post-infectious cerebellar ataxia accounting for 2% of the cases, followed by other inflammatory causes (1%), and PCD (<1%). It has to be noted, however, that no screening for gluten ataxia and anti-GAD-associated ataxia was performed [37]. Concluding, epidemiological data on PCD is sparse and further studies, including prospective population-based studies, are needed to describe the incidence and prevalence of PCD in cerebellar ataxias and populations with different ethnicity.

## 4. Clinical Presentation

Patients with PCD present with a rapidly progressive cerebellar syndrome, which can be preceded by a prodromal phase including fever, headache, nausea, and vomiting. Symptoms at onset typically include a symmetrical limb and truncal ataxia, dysarthria, and nystagmus, although ataxia can be asymmetrical initially in about 40% of patients [15]. Gait ataxia may be the most prominent or sole initial finding, but affection of the trunk and upper limbs, over the course of months, is required to establish the diagnosis of a rapidly progressive cerebellar syndrome [6]. In general, symptoms progress to pancerebellar dysfunction with severe impairment of activities of daily living within three months before stabilizing, leaving most patients wheelchair bound [8,15].

The presence of additional clinical features accompanying cerebellar ataxia are important clinical evidence, as they point to the associated antibody and underlying malignancy. An isolated rapidly progressive cerebellar syndrome is typically associated with Yo-(also known as PCA-1, Purkinje cell antibody 1), DNER-(Tr/delta/notch-like epidermal growth factor-related receptor) and mGluR1-antibodies [8,39,40]. Yo-antibodies are associated with ovarian or breast cancer which can be detected in about 80% of patients. DNER-antibodies associate with Hodgkin lymphoma (detected in 80% of patients) and mGluR1-antibodies associate with hematologic malignancies (detected in about 30% of patients) [22]. Cognitive deficits, however, can be detected in approximately 20% of patients with Yo-antibodies and an otherwise isolated cerebellar ataxia [15]. Symptoms of a more diffuse encephalomyelitis are associated with Hu-antibodies and SCLC, whereas opsoclonus or laryngeal spasms indicate Ri-antibodies and ovarian, breast, or small cell lung cancer [41,42]. When cerebellar ataxia occurs with Lambert-Eaton myasthenic syndrome (LEMS) the presence of VGCC and SCLC should be suspected [11]. It has to be noted, however, that VGCC antibodies can also be present in cerebellar degeneration without an underlying malignancy [43]. Here, SOX-1 antibodies can help to identify patients with an underlying SCLC, as the presence of SOX-1 has a specificity of 100% and a sensitivity of 49% for SCLC [44]. 

## 5. Evaluation

### 5.1. Diagnostic Criteria

In 2004 Graus and colleagues defined diagnostic criteria for paraneoplastic neurologic syndromes [45]. Here, “classical syndromes” that frequently associate with cancer were defined and included encephalomyelitis, limbic encephalitis, subacute cerebellar degeneration, and sensory neuropathy. Furthermore, associated antibodies were divided into “well characterized” and “partially characterized” onconeural antibodies [45]. Based on the clinical syndrome, antibody type, and presence or absence of cancer, two levels of evidence were suggested, which included “definite” and “possible” PNS. Due to new insights in various aspects of PNS in the past 16 years, a group of international experts (PNS-Care panel) updated these diagnostic criteria in 2021 [6]. The term “classical syndrome” was substituted with the term “high-risk phenotype” based on the frequent paraneoplastic etiology of these clinical presentations. Opsoclonus-myoclonus-syndrome, gastrointestinal pseudo-obstruction, and Lambert-Eaton-myasthenic syndrome were added to the list of “high-risk phenotypes”. Furthermore, the term “subacute cerebellar degeneration” was substituted by “rapidly progressive cerebellar syndrome” and the term “onconeural” was replaced by “high-risk”, because not all antibodies are associated with a high risk for cancer development and not all associated cancers express these antigens [6]. Antibodies associated with PNS were thus subdivided into high-risk-antibodies (association with cancer >70%), intermediate-risk-antibodies (association with cancer 30–70%), and lower-risk-antibodies (association with cancer <30%). Based on a scoring system termed PNS-Care Score, three levels of diagnostic evidence for a PNS were suggested, including definite, probable, and possible PNS. The score includes the clinical phenotype, presence or absence of neuronal antibodies and cancer, as well as time of follow-up [6]. For a definite PNS, nowadays, the presence of cancer is obligatory. If cancer was not detected at baseline assessment and high-risk antibodies were identified, cancer screening should be repeated every 4–6 months over a period of 2 years (c.f. Section 5.4). In the context of a rapidly progressive cerebellar syndrome, a definite diagnosis of PCD can be made when a high- or intermediate-risk antibody is found, as well as a tumor consistent with the phenotype and antibody. If the tumor is not consistent with the phenotype of a cerebellar syndrome or the antibody (e.g., gastric adenocarcinoma in a patient with cerebellar ataxia and Yo-antibodies), cognate antigen expression by the tumor has to be demonstrated [6]. Notably, the exclusion of alternative causes is required to establish the diagnosis of a PNS.

### 5.2. Laboratory Testing

#### 5.2.1. Antibodies Associated with Paraneoplastic Cerebellar Degeneration

Identification of autoantibodies in the setting of a rapidly progressive cerebellar syndrome is paramount to (1) establish the diagnosis of PCD, (2) allocate the specific treatment, and (3) predict the association of cancer and direct cancer search [22]. As described in Section 2, antibodies can be classified according to the cellular localization of their target antigens. In this regard, PCD typically associates with intracellular antibodies, but associations with extracellular and synaptic antibodies have been described [1]. Recently, Mitoma and colleagues published a classification for antibodies in IMCAs and suggested two major antibody categories which included (1) antibodies suggestive of specific etiologies and (2) nonspecific autoantibodies found in other neurological and systemic conditions [1]. Additionally, antibodies reported in case reports or case series which were associated with cerebellar ataxia were classified as “not-well-characterized” antibodies [1]. To provide a comprehensive overview on antibodies associated with cerebellar ataxia and PCD in particular and to support clinical diagnosis, we adopted this classification, added antibodies recently described, and complemented it with the classification into high-, intermediate-, and low-risk antibodies according to Graus and colleagues [6]. Here, well-characterized antibodies associated with PCD are summarized in Table 1. Antibodies within this category are “high-risk antibodies” according to Graus et al. and thus represent an important diagnostic factor in the PNS-Care Score [6]. Table 2 lists nonspecific autoantibodies which are present in other neurological conditions but typically present with the additional symptom of cerebellar ataxia. Among this group, “high-risk antibodies” are Kelch-like protein 11- (KLHL11), Purkinje cell cytoplasmic antibody type 2- (PCA2), and Amphiphysin-antibodies, although the presence of Amphiphysin-antibodies is only considered to be a high-risk situation for the association of cancer when polyradiculoneuropathy, sensory neuronopathy, encephalomyelitis, or stiff-person syndrome are present [6]. According to Graus et al., VGCC- and Contactin-associated protein-like 2-antibodies (CASPR2) are associated with an intermediate risk of an underlying tumor. Here, presence of CASPR2-antibodies is considered to be an intermediate risk situation, when the patient presents with Morvan syndrome [6]. “Lower-risk” antibodies comprise dipeptidyl-peptidase-like protein 6-antibodies (DPPX, if encephalitis with CNS hyperexcitability is present), leucine-rich glioma-inactivated 1-antibodies (LGI1, if limbic encephalitis is present), and mGluR1-antibodies (if isolated cerebellar ataxia is present) [6]. Antibodies whose significance must be established but, when detected, can be a clue to the autoimmune etiology of the ataxia are listed in Table 3. When lower-risk antibodies or antibodies whose significance must be determined are found in a patient with a rapidly progressive cerebellar syndrome, probable PCD can only be diagnosed, when antigen expression by a detected tumor can be demonstrated [6].

#### 5.2.2. Antibody Detection

**Figure 1:**
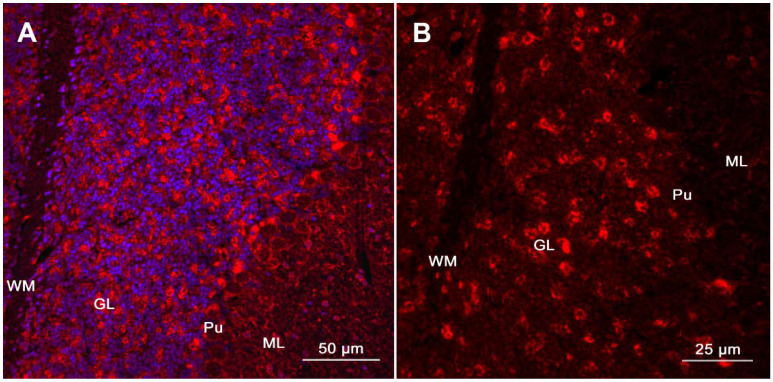
Detection of specific autoantibodies using tissue-based immunofluorescence. (**A**)**.** Immunoreactivity of a patient’s CSF with sagittal mouse brain sections is demonstrated via TIF (Cerebellum, magnification: 100×, counterstaining of nuclei by Hoechst 33342 at 1:10,000). (**B**). Patient’s antibodies show a prominent immunolabeling of the neuropil of the granular layer (GL), whereas binding to the white matter (WM), Purkinje cell layer (Pu), and the molecular layer (ML) is less evident (magnification: 400×). This staining pattern is specific for neurexin-3alpha-antibodies and has been described in the first report of neurexin-3alpha-antibody associated autoimmune encephalitis [108]. Presence of antibodies targeting neurexin-3alpha (a synaptic protein) in patient’s CSF was subsequently confirmed via CBA (Figure adapted from Loehrer et al. [109], reproduced with permission from John Wiley and Sons).

Detection of an antibody in a patient presenting with rapidly progressive cerebellar ataxia is of extraordinary help to diagnose PCD and to determine further management. Gold standard methods for initial screening include immunohistochemistry and immunofluorescence, whereby presence of antibodies is typically detected initially by a technique named tissue-based immunofluorescence (TIF) [22]. Here, sections of brain and non-brain tissue of rodents are incubated with the serum or CSF of the patient tested [22]. Presence of specific autoantibodies can be demonstrated by the application of a second anti-human-antibody. This second antibody binds to the autoantibody and is tagged with a fluorescent label that emits upon photoexcitation [22]. Thus, different staining patterns can be detected, which are characteristic for an antibody (e.g., diffuse neuropil staining specific for neurexin-3alpha-antibodies, see Figure 1). Confirmatory studies employing immunoblot (IB, for most antibodies directed against intracellular proteins) or cell-based assays (CBA, for most antibodies directed against cell surface or synaptic proteins) are employed subsequently [6,22]. As sensitivity and specificity of these techniques varies depending on the sample (serum or CSF) and the antibody tested, it is recommended to perform antibody testing in both, serum and CSF [6]. This is particularly important when suspicion for antibodies against neuronal surface antigens is raised. When neuronal surface antibodies are detected in serum only (and not in CSF), confirmatory TIF or a re-examination in a research laboratory should be pursued before a definite diagnosis is made [6]. 

To standardize approaches in antibody testing and increase diagnostic reliability, recommendations for antibody testing have been published by the PNS-Care panel. These recommendations include the following: testing in serum and CSF (especially for antibodies against surface antigens); focused testing to reduce false-positive and false negative results; consideration of IgG antibodies only (disregard IgA, IgM antibodies as biomarkers); reevaluation in a reference laboratory when antibodies against surface antigens are positive in serum but negative in CSF; the use of above mentioned gold standard methods (TIF, CBA, IB); critical evaluation of incongruences between positive antibodies and neurologic symptoms or cancer; reexamination in research laboratories, when negative antibodies in patients with highly suspicious PNS occur [6]. 

#### 5.2.3. Cerebrospinal Fluid Analysis

CSF analysis typically shows pleocytosis, elevated proteins, and intrathecal synthesis of IgG [8,20]. Absence of inflammatory signs, however, has been reported in case reports and frequency might decrease with age and depends on the associated antibody [110,111,112]. In a study on 155 patients with antibody-associated CNS-syndromes (not specific for PCD) aged 60 years and older, 22.6% did not show signs of inflammation in CSF analysis [112]. When considering seronegative patients, i.e., patients with PCD and without detection of onconeural antibodies, CSF abnormalities could be detected in 88%. Importantly, the frequency of oligoclonal bands was significantly lower in seronegative versus seropositive patients (52% vs. 80%, *p* = 0.03) [21]. It is of note that some patients with PCD show 14-3-3 protein elevation in the CSF, detected by immunoblotting, which could raise the suspicion of Creutzfeldt–Jakob disease (CJD) [113]. In a study of 80 patients with PNS, 12.5% showed positive staining for 14-3-3 protein. The immunoblots, however, showed a double-band pattern in 90% of patients positive for 14-3-3 protein and PNS, whereas a single-band pattern was observed in CJD patients [113]. Therefore, it has been suggested that 14-3-3 protein in the setting of PNS and PCD reflects excessive CNS damage rather than CJD [113].

### 5.3. Imaging Studies

Magnetic resonance imaging (MRI) represents the gold standard of imaging in patients presenting with cerebellar ataxia, while computed tomography has a limited role [114]. MRI findings depend on the phase of disease. While in acute PCD, MRI can be normal or shows T2-hyperintensity of the cerebellar hemispheres, chronic disease often shows cerebellar atrophy, which is best seen in T1 sequences [114,115,116]. Case reports of additional imaging findings associated with specific antibodies have been described. In reports of a patient with Hu- or Yo-antibodies, MRI showed rather diffuse white matter lesions [117,118,119] and diffuse leptomeningeal enhancement of both cerebellar hemispheres. Furthermore, diffuse swelling and slight hyperintensity of cerebellar folia, mimicking acute post-infectious cerebellitis, has been described in a patient with Hodgkin lymphoma and PCD [120]. 

In line with MRI findings, 18F-fluorodeoxyglucose positron-emission tomography (FDG-PET) reveals cerebellar hypermetabolism in acute [121,122,123] and cerebellar hypometabolism in chronic PCD [122].

### 5.4. Cancer Search

Tumors typically associated with PCD are lung cancer (SCLC), gynecological cancers (breast and ovarian cancer) in women, genitourinary cancers (testicular) in men, thymoma, and Hodgkin lymphoma, whereby an association with other tumors (NSCLC, non-Hodgkin lymphoma, prostate cancer, neuroendocrine bladder cancer, Merckel cell tumor, gastric cancer, malignant mesothelioma) have been reported [21]. In up to 70% of the patients with PCD, cerebellar ataxia is the first manifestation of a neoplasm [18] and in 62% of seronegative patients with PCD, symptoms preceded the diagnosis of a tumor with a median time of three months [21]. These findings highlight the necessity for a systemic evaluation for an occult malignancy when patients present with a rapidly progressive cerebellar syndrome. Strong associations between this neurological syndrome, specific antibodies, and cancer type exist (please refer to Table 1, Table 2 and Table 3) and should guide the tumor screening [8]. In 2011 the European Federation of Neurological Societies (EFNS) Task Force published guidelines for cancer search when a PNS, including PCD, is suspected [124]. When screening for a malignancy in the thoracic region (lung cancer, thymoma), a CT-thorax is recommended, which if negative should be followed by an FDG-PET. Breast cancer should be screened for by mammography followed by MRI and FDG-PET, if negative. When screening for a malignancy of the pelvic region or testicular tumors, ultrasound is recommended, followed by a CT-scan, which can be complemented by a FDG-PET [124]. If a patient presents with a rapidly progressive cerebellar syndrome and no antibodies are found, screening by conventional methods (CT-Thorax and ultrasound of the pelvic region) should be performed. In case of a negative screening, a whole-body FDG-PET is recommended [124]. 

If a tumor, which is not consistent with the clinical phenotype and antibody, has been detected, cancer screening should be continued because of the possibility of dual pathology. Furthermore, antigen expression by the tumor should be demonstrated [6]. If the initial screening is negative, the EFNS task force recommended to repeat the screening 4 months after the initial assessment in patients with rapidly progressive cerebellar ataxia and the presence of paraneoplastic antibodies followed by a screening every 6 month up to 4 years [124]. The PNS-Care Panel recommended a screening depending on the detected antibody and clinical phenotype: patients with high-risk phenotype and high-risk antibodies (or intermediate-risk antibodies and additional risk factors, e.g., smoking) should undergo investigations every 4–6 months for 2 years. Patients who do not fulfill these criteria should undergo an extensive screening at initial presentation and rescreening should be considered if patients are refractory to treatment or relapse [6]. Importantly, these are general recommendations that have to be adapted individually.

### 5.5. Differential Diagnosis

When patients present with cerebellar ataxia, initial differential diagnoses are wide [8]. Although metastasis, cerebrovascular, and demyelinating disease can be detected by MRI, it can be more difficult to differentiate toxic, metabolic (e.g., vitamin deficiency (B12, B1, E), hypothyroidism), infectious/postinfectious (HIV, CJD, Miller-Fisher-Syndrome), autoimmune (e.g., GAD-associated-syndromes, PACA (primary autoimmune cerebellar ataxia), and degenerative etiologies [8]. For newly discovered antibodies with unknown clinical significance, differentiation between PCD and PACA can be challenging. Diagnostic criteria for PACA include a predominantly subacute or acute cerebellar syndrome, a MRI which is normal or shows cerebellar vermian atrophy, and two of the following criteria: CSF pleocytosis and/or positive IgG CSF restricted oligoclonal bands, autoimmune disorders in the patient’s history or family history, and detection of an autoantibody that supports autoimmunity but has not yet shown to be directly involved in the pathogenesis of ataxia [73]. Furthermore, alternative causes must be excluded [73]. Toxins that cause cerebellar damage are alcohol, carbon tetrachloride, heavy metals, phencyclidine, thallium, and toluene. Medications associated with cerebellar ataxia include antibiotics/virostatics/antihelminthics (metronidazole, piperazine, zidovudine), antiepileptics drugs (phenytoin), sedative drugs (barbiturates, benzodiazepine, bromides), chemotherapeutic agents/immunosuppressive drugs (asparaginase, cyclosporine, cytarabine, fluorouracil, tacrolimus) and others (amiodarone, bismuth, glucocorticoids, lithium; this list is not exhaustive) [8,125]. Therefore, thorough medical history taking, laboratory testing (including vitamin levels, thyroid function tests, HIV serology, anti-gliadin, and anti-GAD-antibodies), and CSF analysis (as mentioned above) are important to establish the diagnosis of PCD. Furthermore, genetic testing should be considered in the appropriate clinical setting. As described above, Hadjivassiliou et al. reported prevalence-levels of different etiologies of progressive ataxias within a large single center cohort in the United Kingdom [2]. Among sporadic ataxias, following etiologies were reported (numbers in brackets represent percentage out of total sporadic cases): gluten ataxia (25%), genetic cause (13%, without family history), alcohol-related (12%), multiple system atrophy-cerebellar type (11%), myoclonic ataxia (3%), paraneoplastic cerebellar ataxia (3%), anti-GAD-associated ataxia (2%), phenytoin-related (2%), cerebellitis (1%), superficial siderosis (1%), opsoclonus-myoclonus ataxia, episodic ataxia (negative genetics), ataxia with palatal tremor, HIV-related, and Wernicke’s disease (each <1%) [2].

## 6. Treatment and Management

Therapeutical approaches have to be distinguished between symptomatic therapy, acute and maintenance immunotherapy, and treatment of the underlying oncologic diagnosis in terms of curative or palliative therapies. Therapeutical approaches of symptoms (e.g., ataxia, nystagmus, psychological symptoms) are not different from standard treatment of these symptoms due to other diseases and are therefore not subject of this review. Treatment strategies have to take into account the detection of autoantibodies, underlying conditions, and current state of the disease. Due to the rarity of cases, current data lack the evidence of large randomized clinical trials. Therefore, therapeutical approaches are mainly based on (supposed) pathophysiology, case reports, and clinical experience.

### 6.1. Oncologic Treatments

In most cases, treatment of the underlying oncological disorder is paramount for the treatment of the paraneoplastic syndrome as well. An effective, early oncologic treatment can lead to treatment of the paraneoplastic syndrome by reducing the autoimmune driving force by reduction of antigen presentation [18,126,127]. These effects cannot be expected to develop immediately, and thus oncologic treatment does not substitute acute immunotherapy in most cases. Early and effective oncologic treatment, therefore, should be prioritized and is associated with overall survival from an oncologic viewpoint as well as with better treatment responses and neurological outcome [128]. Of course, choice of oncological treatment options (surgery, chemotherapy, radiotherapy) depends on tumor entity, staging, and individual aspects [127]. Unfortunately, neurological symptoms may develop slowly, precede other symptoms, or may be misdiagnosed as other neurological disorders delaying correct diagnosis and treatment until a substantial neurological damage has occurred.

### 6.2. Acute Immunotherapy 

If a patient presents with a typical clinical syndrome and specific antineuronal antibodies are detected or suspected, acute therapeutical settings aim to reduce brain inflammation and levels of circulating antibodies. 

In most cases, corticosteroids are chosen as first-line approach due to their easy administration, wide and fast availability, rare acute adverse effects (mostly hyperglycemia and psychosis), and few strict contraindications. Intravenous methylprednisolone should be chosen in a dosage of 1000 mg daily for 3–5 days. Courses may be repeated. Equivalent oral administration of prednisolone may be considered but in general exhibit a higher risk of adverse effects. From a pathophysiological point of view, corticosteroids target brain inflammation, edema, and disruption of the blood-brain barrier on the one hand and lead to apoptosis of antibody-producing plasma cells on the other hand [129,130]. 

Reduction of circulating autoantibodies can also be addressed by intravenous immunoglobulins (IVIG) [131] or plasma exchange (PLEX) [132]. Both strategies can be used as add-on therapy to corticosteroids in severe cases or as a second-line approach in case of lacking therapeutical effects of corticosteroids. The choice between IVIG and PLEX is mostly based on availability and possible adverse effects. IVIG are contraindicated in patients with IgA deficiency and severe kidney failure and have to be used carefully in patients with acute or chronic heart failure and in thrombogenic states [133]. They are easy to administer in a dosage of 0.4 g/kg daily for 5 days and are widely available. PLEX is in need of invasive high-volume central line placement, may induce or aggravate hypotension, and may be difficult or not possible due to its need of (temporary) anticoagulation, mainly with unfractionated heparin. PLEX is usually performed for 5–7 exchanges. Therapeutical effects of both regimen (IVIG or PLEX) seem to be of equivalent efficiency, although randomized clinical trials in antibody mediated paraneoplastic syndromes are still missing. Many case reports [134,135] and clinical experience show efficacy of both regimens. Since good data for IVIG and PLEX is available in other antibody-mediated peripheral and central entities such as Guillian-Barré-syndrome and non-paraneoplastic autoimmune encephalitis [136,137], one can extrapolate the potential benefit of IVIG and PLEX based on pathophysiological considerations.

In summary, we recommend intravenous methylprednisolone (1000 mg/d for 5 days) as first-line treatment. Addition of either IVIG or PLEX should be considered simultaneously in patients with severe symptoms or rapid clinical worsening. In case of missing effect of monotherapy with corticosteroids, IVIG or PLEX should be initiated.

### 6.3. Maintenance Immunotherapy

Maintenance immunotherapy is initiated either to maintain and enhance positive effects of the initial immunotherapy or to prevent relapses. Oral corticosteroids are mostly used as a bridging concept in case of good clinical response to first-line therapy with methylprednisolone. Tapering regimens beginning with a dosage of 1 mg/kg prednisolone are mostly used.

If a specific antibody has been detected, one can distinguish between antibodies directed against intracellular antigens and antibodies directed against cell-surface antigens. In the first case, regimens targeting T-cell based mechanisms might be of advantage. Therefore, regimens targeting B-cells alone (e.g., anti-CD20 antibody rituximab) might not be as effective in these cases. Concepts targeting B-cells alone are primarily used if antibodies against cell-surface antigens have been detected. In the case of an unknown target antigen, prediction of the effectiveness of either strategy may not be possible ex ante. According to a recent survey, most therapist chose rituximab over cyclophosphamide in a setting with a supposed and unknown antibody [138]. 

Strategies targeting all types of immune cells (mainly T- and B-cells) include substances such as azathioprine (at a daily dosage of 2–3 mg/kg), mycophenolate mofetil (2000 mg/d) [139], and cyclophosphamide (975 mg/m² intravenous, monthly) [140]. Azathioprine and mycophenolate mofetil are oral inhibitors of purine synthesis and need several weeks to establish their clinical effectiveness. Cyclophosphamide induces apoptosis and leads to effective and fast immunosuppression. 

In case of intended depletion of CD20 positive plasma cells, rituximab is a well-tolerated substance. Regimens typically include infusions of 1000 mg on day 0 and 14 and are repeated every 6 months. Effectiveness and dosage intervals can be monitored by B-cell count. Especially after good clinical response by PLEX, even in the absence of a specific antibody, this is a feasible therapeutical approach.

In case of contraindications or adverse effects under therapy with immunosuppressants, monthly administrations of IVIG or performance of PLEX can be considered in the presence of good clinical response.

## 7. Outcome and Prognosis

Overall prognosis of paraneoplastic neurologic syndromes and PCD in particular is poor [1,17,18,127,141,142]. Early detection and treatment of the underlying neoplastic condition is, as a matter of course, the main predictor of overall survival [47]. For example, analysis of 50 patients with antibody positive PCD showed that antitumor treatment may result in complete remission [17]. The functional outcome was best in Ri-antibody positive patients, but only 4/19 patients with Yo-antibodies and 4/16 patients with Hu-antibodies remained ambulatory. Median survival differed between 7 months (anti-Hu) and >113 months (anti-Tr) [17]. The therapeutic effect of acute and maintenance immunotherapy is often scarce. Many patients show a rapid progression of symptoms over the initial weeks and may stabilize under therapy on low level. Detection of cerebellar atrophy in the early course or at the time point of stabilization predicts a low prognosis regarding substantial improvement. An older study on 22 patients presenting with cerebellar degeneration due to anti-Yo showed <10% clinical improvement under treatment regimens with PLEX, corticosteroids, and cyclophosphamide [15]. Different case series reported better clinical outcome when immunotherapy was administered in the very early course of the disease [143,144]. 

Paraneoplastic cerebellar degeneration often results in a rapidly progressive and devastating neurological course. Treatment should be started as early as possible before pronounced cerebellar degeneration has taken place. It is paramount, that absence of a specific antibody is not equated with absence of autoimmunity and therefore should not prevent early immunotherapy. Especially, acute immune-therapeutical approaches such as corticosteroids, IVIG, or PLEX have a moderate probability of adverse effects and may therefore be used even in situations when a definite diagnosis has not been established.

## 8. Conclusions

Paraneoplastic cerebellar degeneration is a rare but devastating disease. Diagnosis and management of patients with PCD requires detailed knowledge and an interdisciplinary approach. Newly identified antibodies associated with a rapidly progressive cerebellar syndrome have been described in small numbers of patients, making it difficult to estimate clinical significance and broadening differential diagnosis. Recently published diagnostic criteria help to establish a diagnosis of PCD and to guide tumor screening as well as treatment approaches. General treatment approaches, primarily based on immunotherapy and oncologic treatment, exist but lack evidence. Therefore, several aspects have to be addressed in future research: (1) the significance of recently described antibodies associated with a rapidly progressive cerebellar syndrome has to be clarified and possible pathogenic effects of these antibodies detected, (2) targeted treatments for antibody mediated PCD should be developed, and (3) large-scale, multi-center, multi-national studies are needed to evaluate different treatment options and prognostic factors.

## Figures and Tables

**Table 1:** Well-characterized antibodies in PCD.

Antibody Target	Neurologic Phenotype	Gender Predominance, Age-Range	Associated Cancer	Frequency of Cancer	Source
Intracellular target					
CV2/CRMP5	EM, SNN, CA	No gender predominance, Age: 60–70	SCLC, thymoma	>80%	[46,47,48]
Hu (ANNA-1)	SNN, CA, EM, LE, chronic gastrointestinal pseudo-obstruction	Slight female predominance, Age: 60–70	SCLC > NSCLC, neuroendocrine tumors, neuroblastoma	85%	[22,49]
Ma2	LE, diencephalitis, CA, brainstem encephalitis	70% male, Age: 60–70 in women, 30–40 in men	Testicular cancer (young men), lung cancer (older patients)	>75%	[50,51]
Ri (ANNA-2)	CA, OMS	Female predominance, Age: 60–70	Breast (women) > lung cancer (men)	>70%	[22,42,52]
Yo (PCA-1)	CA	Almost all female, Age: 60–70	Ovary and breast cancer	>90%	[22,40]
Extracellular target					
TR (DNER)	CA	>70% men, Age: 60–70	Hodgkin lymphoma	90%	[39,53]

Table adopted from Mitoma et al. and Graus et al. [1,6]. **Abbreviations: CV2/CRMP5**: collapsin response-mediator protein 5, **Hu (ANNA-1)**: antineuronal nuclear antibody-1; **Ma2**: metabotropic glutamate receptor2; **Ri (ANNA-2)**: antineuronal nuclear antibody-2; **Yo (PCA-1)**: Purkinje cell antibody; **TR (DNER)**: delta/notch-like epidermal growth factor–related receptor; **CA:** cerebellar ataxia; **EM:** encephalomyelitis; **LE:** limbic encephalitis; **NSCLC:** non-small-cell lung cancer; **OMS:** opsoclonus myoclonus syndrome; **SCLC:** small-cell lung cancer; **SSN:** subacute sensory neuronopathy.

**Table 2:** Antibodies associated with cerebellar ataxia and additional neurologic syndromes.

Antibody Target	Neurologic Phenotype	Gender Predominance, Age-Range	Associated Cancer	Frequency of Cancer	Source
Intracellular target					
Amphiphysin	Polyradiculo-neuropathy, SNN, EM, SPS, CA	Slight female predominance, Age: 60–70	SCLC, breast cancer	80%	[54,55]
GAD65	LE, SPS, CA	70% women, Age: 50–60	SCLC, neuroendocrine tumors, thymoma	<15%	[56,57]
KLHL11	CA, brainstem syndrome	100% men, Age: 40–50	Testicular cancer	80%	[58,59,60]
MAG *n* = 5	Neuropathy, CA	100% men, Age: 60–80	Unknown, MGUS association	Unknown	[61]
PCA2 (MAP1B)	Limbic/brainstem encephalitis, LEMS, SIADH, Neuropathy, CA (37% of reported cases)	Female predominance, Age: 22–89	SCLC, NSCLC, breast, renal, skin squamous cell, pancreas, extrapulmonary small-cell, prostate, intrahepatic primary ductal, nasopharyngeal, Ewing sarcoma, lymphoma	80%	[62,63]
Extracellular target					
CASPR2	LE, Isaac syndrome, Morvan syndrome	>70% men, Age: 60–70	Thymoma	<30%	[64,65,66]
DPPX	Encephalitis, CNS hyperexcitability, PERM	>60% men, Age: 50–60	B-cell malignancies	<10%	[67,68]
LGI1	LE, CA	>60% men, Age: 60–70	Malignant thymoma, neuroendocrine tumors	<10%	[22,69,70]
mGluR1	CA, dysgeusia	No gender predominance, Age: 50–60	Hematologic	20–30%	[22,71]
P/Q VGCC	LEMS, CA	Slight female predominance, Age: 50–60	SCLC	50% (LEMS), 90% (in pts. w/CA)	[11,72]

Table adopted from Mitoma et al. and Graus et al. [1,6]. **Abbreviations: CASPR2:** contactin-associated protein-like 2; **DPPX:** dipeptidyl peptidase-like protein; **GAD65**: glutamic acid decarboxylase, **KLHL11**: Kelch-like protein 11; **MAG**: myelin-associated glycoprotein; **mGluR1:** metabotropic glutamate receptor 1; **PCA2**: purkinje cell antibody 2; **VGCC:** voltage-gated calcium channel **CA:** cerebellar ataxia; **CNS:** central nervous system; **EM:** encephalomyelitis; **LE:** limbic encephalitis; **LEMS:** Lambert-Eaton myasthenic syndrome; **NSCLC:** non-small-cell lung cancer; **OMS:** opsoclonus myoclonus syndrome; **PERM:** progressive encephalomyelitis with rigidity and myoclonus; **SCLC:** small-cell lung cancer; **SPS:** stiff-person syndrome; **SSN:** subacute sensory neuronopathy.

**Table 3:** New, rarely characterized antibodies of unknown significance associated with cerebellar ataxia.

Antibody Target	Neurologic Phenotype	Gender Predominance, Age-Range	Associated Cancer	Frequency of Cancer	Source
ARHGAP 26 (GRAF1-IgG, Anti-ca) *n* = 24	subacute CA, neuropathy, psychotic symptoms, cognitive dysfunction, hyperekplexia, parkinsonism	No gender predominance, Age: 14–76	Ovarian, Breast, Melanoma, B cell lymphoma, prostate, gastric, squamosa cell of nasopharyngeal/respiratory tract	30–40%	[22,73,74,75]
CARP VIII *n* = 3	CA, headache	Female predominance, Age: 69–77	Ovarian cancer, melanoma, breast	3/3	[76,77,78]
Glycin R *n* = 187	PERM/SPS 40–50%; epilepsy 20–30%; CA, movement disorders, encephalitis (30%)	No gender predominance, Age: 40–60	Thymoma, breast cancer, Hodgkin lymphoma, SCLC, marginal B-cell lymphoma	10–20%	[22,79]
Homer-3 *n* = 5	CA, encephalitis, papilledema	No gender predominance, Age: 38–65	SCLC	1/5	[73,80,81,82,83]
mGluR2 *n* = 2	CA	Female predominance, Age: 3–78	Small cell tumor, alveolar rhabdo-myosarcoma	2/2	[84]
Nb/AP3B2 *n* = 13	CA, peripheral neuropathy, myelopathy	Female predominance, Age: 24–58	Renal cell cancer, B-cell lymphoma	2/13	[73,85,86,87]
Neurochondrin *n* = 14	CA, brainstem, myelopathy, psychosis, SFN	Male predominance, Age: 2–69	Uterine cancer	1/14	[73,88,89,90]
NIF *n* = 41 (11 CA)	Encephalopathy, CA (27%), myelopathy, neuropathy	Male predominance, Age: 43–88	Merkel cell carcinoma, SCLC, neuroendocrine (pancreas), Hodgkin lymphoma, hepatocellular carcinoma	8/11	[91]
PKCy *n* = 10	CA	Male predominance, Age: 47–73	NSCLC, adenocarcinoma of hepatobiliary origin	unknown	[92,93]
Septin-5 *n* = 6	CA, oscillopsia	No gender predominance, Age: 47–72	No association	none	[73,94]
SEZ6L2 *n* = 6	CA, extrapyramidal symptoms, retinopathy	No gender predominance, Age: 54–69	Breast cancer	1/6 (4 year after CA)	[95,96,97]
Sj/ITPR-1 *n* = 23 (11 CA)	CA, polyneuropathy, encephalopathy, myelopathy	No gender predominance, Age: 7–83	Breast, lung, renal, endometrial cancer, myeloma	7/23 1 breast cancer 11 years after CA	[73,80,98,99,100,101]
SOX1 (AGNA1) *n* ≈ 520 (20 PCD)	LEMS (30%), CA (18.2%), limbic encephalitis (18.2%), neuropathy	Male predominance, Age: 17–87	SCLC >> NSCLC>, Hodgkin lymphoma, breast, prostate, thyroid, esophageal cancer	>90%	[102]
TRIM 9, 67 *n* = 3	CA, gaze palsy	No gender predominance, Age: 65–78	Lung cancer, Melanoma	2/2	[103,104,105]
TRIM 46 *n*= 3	Progressive encephalomyelitis, CA, rapidly progressive dementia	No gender predominance	SCLC	2/3	[104]
ZIC4 *n* = 20	CA, OMS, SSN, dementia, SPS, brainstem encephalitis, pain, limbic encephalitis, LEMS	Male predominance	SCLC, B-cell lymphoma, multiple myeloma, breast, ovarian cancer, head and neck squamosa cell carcinoma	14/20	[7,106,107]

Table adopted from Mitoma et al. and Graus et al. [1,6]. **Abbreviations: Ca/ARHGAP26**: Ca/Rho GTPase-activating protein, **CARP VIII**: carbonic anhydrase-related protein VIII; **mGLUR2**: metabotropic glutamate receptor2; **NB/AP3B2**: Nb/adaptor complex 3B2; **NIF**: Neuronal intermediate filament light chain; **SEZ6L2**: seizure-related 6 homolog like 2; **Sj/ITPR-1**: inositol 1,4,5-triphosphate receptor type 1; **SOX-1**: sex-determining region Y-related high-mobility group box 1; **TRIM 9, 67, 46**: tripartite motif-containing protein 9, 67, 46; **ZIC4:** zinc finger protein of the cerebellum 4 **CA:** cerebellar ataxia; **LEMS:** Lambert-Eaton myasthenic syndrome; **NSCLC:** non-small-cell lung cancer; **OMS:** opsoclonus myoclonus syndrome; **PERM:** progressive encephalomyelitis with rigidity and myoclonus; **SCLC:** small-cell lung cancer; **SIADH:** syndrome of inappropriate antidiuretic hormone secretion; **SPS:** stiff-person syndrome; **SSN:** subacute sensory neuronopathy.

## Data Availability

Not applicable.
